# Working on the Frontline of Dog Adoption: The Perspectives and Experiences of Animal Shelter Workers in RSPCA Queensland

**DOI:** 10.3390/ani16081279

**Published:** 2026-04-21

**Authors:** Eileen Thumpkin, Nancy A. Pachana, Mandy B. A. Paterson

**Affiliations:** 1 School of Psychology, The University of Queensland, Brisbane, QLD 4072, Australia; n.pachana@psy.uq.edu.au; 2 Royal Society for the Prevention of Cruelty to Animals Queensland, Brisbane, QLD 4076, Australia; mpaterson@rspcaqld.org.au; 3 School of Veterinary Science, The University of Queensland, Gatton, QLD 4343, Australia

**Keywords:** rescue shelter workers, dog adoption, post-adoption support, animal shelter, focus group, intervention, meet-and-greet

## Abstract

Estimates suggest that approximately 400 million dogs are kept as pets worldwide. Despite their popularity, around 10% to 30% are surrendered to rescue shelters each year. Shelter workers play a pivotal role in the success of dog adoptions and provide ongoing support to help owners keep these dogs in their homes. However, research that captures their perspectives and experiences regarding the dog adoption process remains limited. Royal Society for the Prevention of Cruelty to Animals Queensland shelter teams participated in six focus group discussions to share their perspectives and experiences of the dog adoption process in their shelters. Reflexive thematic analysis of the gathered data generated three themes: 1. “Doing great adoptions” starts with an inclusive, well-resourced application process and a skilled team. 2. Finding the right fit involves navigating the duality of carer and advocate through honest, informative interactions with the whole family. 3. Successful outcomes involve supporting and educating the public to care for and keep their dog. This grounded understanding of the challenges facing shelters in their work could provide valuable feedback to help shelter leaders and staff develop policies and practices that support positive adoption outcomes, tailor programmes to local needs, and reduce return rates.

## Simple Summary

Improving dog adoption outcomes is challenging for animal rescue organisations. Shelter workers are the vital link between the dogs in their care and prospective adopters. This study visited shelters to explore staff perspectives and experiences regarding the dog adoption process. Most participants agreed that the application was the first step in the process, followed by an interview with shelter staff and then a meeting with the dog. Finally, although post-adoption support was limited in most shelters, participants agreed it was important in helping adopters keep their dogs in the home.

## 1. Introduction

Dogs occupy a unique place in the world. It is said that “they are stitched into humans’ hearts” and the fabric of daily life [1] (p. 28). Acquiring accurate statistics on dogs kept as companion animals worldwide is challenging, as is the accuracy of numbers surrendered to rescue organisations. However, estimates suggest that globally there are around 400 million dogs kept as pets [2,3,4,5]. Dogs remain the most popular companion animal, playing many different roles in people’s lives—roles that have “changed dramatically over the past two centuries” from working dog to companion; they are an essential part of many people’s lives [6] (p. 1). Discussions in the public sphere, information promoted by the pet industry, communications from animal welfare organisations, and published research increasingly position dogs as family members [7,8,9,10,11]. Despite this ongoing popularity, data estimates suggest that between 10% and 30% of pet dogs—equivalent to 12 to 15 million—are surrendered to shelters each year [5,12,13,14,15,16]. These figures do not include the millions of stray dogs that also enter shelters in need of care and homes [17].

Shelters play a vital role in improving the life outcomes of these animals, and this role continues to evolve in response to changing community attitudes and expectations regarding animal care and protection [18] (p. 6), [19]. Today, in addition to managing animal care and rehoming, shelter organisations play an essential role in many communities by providing education and outreach and by partnering with other service providers to support people in need and their companion animals [18,20,21]. The shelter staff and volunteers working on the frontline of animal care and adoption are a relatively unexplored source of data on contemporary shelter care and adoption processes, offering valuable insights into improving animal outcomes.

Although an extensive body of multidisciplinary qualitative and quantitative research exists on animal rescue organisations, companion animals, adopters, and workers involved in animal sheltering [22], much of this work has focused on identifying factors that influence the decision to acquire, retain, or relinquish a companion animal [23,24,25,26,27]. Additionally, research has investigated factors that improve dogs’ adoption prospects, including dog characteristics, behaviour and training, fostering, matching programmes, and organisational approaches to adoption policy and processes, such as closed and open adoption [28,29,30,31,32]. This research aims to enhance the welfare and life outcomes of animals in care, support the wellbeing of shelter workers, and help people build strong, lasting bonds with their dogs. A central focus of shelter worker research has been the psychological impacts of exposure to euthanasia, resourcing constraints, the demands of the job, the acceptance of surrendered animals, exposure to cruelty, and the management of public expectations and distress [33,34,35,36,37,38]. Irvine aptly captures the complex nature of their daily work:

*On any given day, shelter workers might experience intense joy at seeing an animal placed in a loving home, profound anger at instances of cruelty, deep sadness during euthanasia, and shock or horror at the ignorance people have about animals—all with little opportunity to express or act on their feelings.*

[33] (p. 83).

These workers are vital to the daily care and rehoming of shelter animals, as well as to providing ongoing support to owners. They serve as the link between animals, adopters, and the community. Although there is extensive multidisciplinary research on animal sheltering, much of it relies on surveys, scales, and shelter data. In contrast, research on how to utilise shelter workers’ insights to improve their daily work, offer better support, and refine policies and processes remains limited [39] (pp. 7–8). There is little research that places the staff’s voice at the centre of data collection to understand the nuances of how they shape their daily tasks to manage competing demands, stresses, and challenges in facilitating animal adoption [33,36,37,40].

### Current Study

This study aimed to explore the adoption processes at the Royal Society for the Prevention of Cruelty to Animals, Queensland (RSPCA Qld), from the perspective of shelter workers, to gain a more comprehensive understanding of the complex interactions between staff and adopters. The COVID-19 pandemic significantly affected shelter organisations, resulting in substantial changes to their adoption processes. In a 2023 study, Carrol et al. identified three main challenges faced by animal shelter organisations: the impacts on animals, organisational identity, and operational procedures [41] (p. 1). The latter is particularly relevant to the current study, as COVID-19 notably influenced adoption practices within RSPCA Qld shelters.

Prior to the pandemic, potential adopters could view all available dogs online. At that stage, it was not possible to express an interest in adopting a dog; adopters were required to come into the shelter. However, when shelters had to close their doors from 18 March to 27 June 2020, an online model was developed. The animals were presented in different formats, including videos, photographs, and information blurbs. These were designed to speak in more detail to people because the adoption was almost entirely virtual. Adopters and dogs met for the first time during pickup from the shelter. The impacts of this COVID-19 companion animal adoption phenomenon have been the focus of several research projects [42,43,44,45]. Post-pandemic, RSPCA Qld shelters adopted a hybrid model that involved online selection and in-shelter meet-and-greets.

Ultimately, this study aimed to explore the challenges faced by shelter staff and to gather their insights and experiences regarding dog adoption processes and efforts to improve long-term outcomes, which remain a challenge for shelters. Gaining a deeper, on-the-ground understanding could provide valuable feedback to help shelter leaders and staff develop policies and practices that support positive adoption outcomes, tailor programmes to local needs, and reduce return rates.

Using an interpretivist, qualitative approach allowed the researchers to explore a range of perspectives and experiences and to gain a more granular understanding of shelter staff’s daily work across various geographical locations, communities, and shelter settings. Braun and Clarke [46], however, caution researchers that “*pure* induction is impossible; the researcher always brings philosophical metatheoretical assumptions and themselves to the analysis, meaning an inductive orientation is better understood as ‘grounded’ in data” [46] (p. 6). An inductive approach is hypothesis-generating; it is not directed by preconceived ideas of what will be found in the data [47,48].

Visiting shelters, participating in group discussions with staff and volunteers, and listening to their insights provided us with rich, detailed data on their experiences and perspectives regarding the adoption process in the field. The focus group environment fostered interactive discussion that, as Hennink writes, “prompts rationalisations, explicit reasoning, and focused examples, thereby uncovering various facets and nuances of the issues that are simply not available by interviewing an individual participant” [49] (p. 3).

Many qualitative researchers emphasise that engaging with participants is an interpersonal, subjective process involving both the researcher and the participants, as each brings their own experiences, values, perspectives, assumptions, and interests in the phenomenon under study [50,51,52,53]. Importantly, researchers must be actively aware and critically reflexive of how “their interpretation (their analysis) of what they see, hear, and experience during research is informed by” their positionality, which includes their social identity, background, and worldview [52] (p. 707), [54,55,56].

The authors recognise that researcher positionality influences how qualitative data are collected and interpreted [47,50,57]. The research team brings diverse disciplinary backgrounds, including Psychology, Sociology, Education, Veterinary Medicine, and Animal Welfare. All three have experience living with adopted companion animals; at the time of this study, one researcher lived with cats, another with cats and birds, and the third with dogs. Each maintains ties to RSPCA Qld. Researcher MP has served as Principal Scientific Officer for RSPCA Qld, and all three have volunteered as foster carers and adopted animals from the organisation. ET was a former board member of RSPCA Qld and RSPCA Australia. MP and ET were both mindful of the possible impact on participants’ willingness to share honestly, given our connections to executive personnel. However, MP was not involved in the focus groups, as ET was the primary study contact and moderator. During discussions with shelter staff, ET explained that this was an independent study as part of her doctoral programme, emphasising that the focus was on capturing staff insights as an invaluable source of information about dog adoption processes in their shelters. The moderator exercised care to stay within the study’s scope and to check herself for bias, avoiding drawing on her perspectives or assumptions. Following each session, she also reflected on the structure, participation, timing, and her moderation, noting any personal bias during the discussions and adjusting where required in future groups and analyses.

## 2. Materials and Methods

Six focus groups were held to gather data from the shelter teams involved. These discussions generated a range of insights, perspectives, and detailed examples of the challenges encountered in managing dog adoption processes in their shelters. At the time this research was conducted, RSPCA Qld operated nine shelters across the state. These centres, each serving a distinct community, differed considerably in size, capacity for sheltering and animal care, and the range of on-site services available beyond basic care, such as behaviour training, staffing, and volunteer numbers.

Although focus group discussions are an efficient method of collecting data, and they can support participants to engage, they may also “dampen disclosure, reduce the diversity of opinions, encourage the endorsement of each other’s views, or pose challenges for researchers committed to protecting anonymity” [57] (p. 310). In this study, the researchers were mindful of the importance of participant confidentiality and of encouraging open discussion.

The RSPCA Qld CEO and Board approved the research project; however, they had no financial or advisory role in the planning or implementation of this study.

### 2.1. Participant Recruitment

Initial contact was made via email and phone with all nine RSPCA shelter managers to gauge their interest in participating in the study. An offer was also made to provide an online briefing for staff about the study. Following these conversations, a follow-up email was sent to all shelter managers and dog adoption coordinators, including copies of the participation information and a brief background on the study. Over the following months, researcher 1 (ET) followed up with the nominated shelter contacts to clarify any issues and tentatively organise the focus group sessions at their respective shelters. Participants also had the option to provide confidential responses to the topics covered if they were unable to attend a focus group. This option was available for those who might not have been at work on the day or who felt uncomfortable in a group setting.

Initially, all nine shelters expressed interest in the study; however, six ultimately participated in the focus groups. Shelters are busy places, and some meetings were rescheduled several times to accommodate staffing schedules, availability, and workloads. One participant, who was unavailable to join the focus groups, requested a phone interview to discuss the topics.

### 2.2. Focus Group Sessions (Data Collection)

Researcher 1 (ET) conducted six in-person focus group sessions and one phone interview between November 2024 and February 2025. Each session was held at the shelter, with group sizes ranging from 3 to 12 participants. There were 37 participants in total. Most participants were female staff (89%), and most had worked in shelters for less than 10 years. The smaller groups reflect the shelters’ staffing and resourcing levels. Volunteers were invited to participate; however, only four were involved. Several shelters reported difficulty attracting volunteers. Meetings were scheduled around morning tea or lunch breaks. The researcher provided the lunches or morning teas. This approach facilitated initial conversations to build rapport with participants, answer questions, and introduce the researchers. Participants were assured that ET was no longer involved with the RSPCA Boards and that the study was an independent doctoral study. The only information shared with the organisation would be provided in a published paper or, upon request, in a presentation by the researchers.

Participants were reassured that the shelter’s identity, specific location, and their personal details would not be included in research papers or presentations. All recordings were accessible only to the three researchers, and the transcripts were de-identified, with place names and participant details replaced with letters and numbers. Furthermore, care would be taken to ensure that any quotes used would not reveal the location or identity of any participant. ET was very aware of the potential influence of organisational politics and sensitive to participants’ concerns about sharing their perspectives. It was reiterated that the focus group topics concerned dog adoption processes in their shelters; it was not a critique of the broader organisation or its staff. Naturally, there would be some tangential references to related RSPCA Qld policies and procedures.

Before commencing the session, the focus group process was outlined, including the three broad topics to be considered. The consent forms were distributed for initial signature, and participants were asked whether they consented to the session being recorded. Participants were also reminded to maintain the confidentiality of discussions during their session (the focus group discussion guide and questions are provided in Appendix A). The three broad topics guiding the discussion were:

1. Adoption application processes and the impact of COVID-19 on these processes;
2. The adoption interview and the procedure for taking the dog home;
3. Post-adoption follow-up and support.

Discussions took place in various settings. In smaller shelters, they occurred in a small office or staff area near the reception desk, or outdoors close to the shelter’s reception point. One session was held in a designated meeting room. Holding these sessions at the shelters provided context—a brief insight into daily life there—and enabled observation of team dynamics. They offered a real-time view of the public interface, meet-and-greet procedures, and daily care routines. Shelters are busy environments, and it was recognised that interruptions might happen if staff were called away; recordings would be paused and resumed as needed. Sessions lasted between 45 and 80 min. Participants generally stayed engaged, reflective, and insightful throughout the discussions.

At the end of each session, a monetary donation was given to the staff social club as a token of appreciation for their participation.

### 2.3. Data Analysis

Researcher 1 led the reflexive thematic analysis following Braun and Clarke’s [51,58,59] six-phase process, recognising it as an iterative process. This is a theoretically flexible approach that ensured our analysis was grounded in and shaped by the voices of shelter staff recounting and discussing their experiences of dog adoption processes in their shelters [60,61].

Following each focus group session, ET transcribed verbatim the digital recording into a Word table organised under the three topic questions. Focus group participants were identified in the transcripts as P1, P2, and P3, in the order in which they spoke during the session. Each session was noted as FG1–FG7 (the single interview conducted was labelled FG7), with any mention of a specific location referred to generically as our shelter or our community. Once completed, each transcript was checked for accuracy against the recording and reread, with initial ideas noted and illustrative text highlighted, allowing a deeper level of familiarity with the dataset (Phase 1). During Phase 2, initial descriptive and latent code labels were noted in the Word tables. Once the dataset transcriptions were completed, responses from all sessions were collated into a single Word table, organised under the three discussion topics, with accompanying columns for potential codes, themes, and illustrative quotes. On re-reading, codes were clustered, and potential themes were developed and reassessed to ensure they reflected participants’ stories about the dog adoption processes and captured the recurring patterns in the dataset (Phase 3).

At this stage, the three researchers discussed the candidate themes to determine whether they were consistent and could present a coherent account of the adoption process across the dataset. After review and refinement, three themes were identified. In the final stage of writing up the themes, it was evident that they could be interpreted at several levels: the individual, the team or shelter collectively, and the organisational level (Phases 4–6).

## 3. Results

Three themes were generated from the thematic analysis of data on dog adoption processes across the six shelters. These are: 1. “Doing great adoptions” starts with an inclusive, well-resourced application process and a skilled team. 2. In negotiating the right match, the meet-and-greet is the vital step. 3. Successful outcomes involve supporting and educating the public to care for and keep their dog.

These themes are interrelated, and common concepts recur throughout the dataset, as staff recounted their efforts to “set the adopters and the dogs up for success” while balancing their dual roles as protectors and agents responsible for finding a good home for the dogs Reese [62]. They illustrate how shelter staff negotiate the complexities of shelter life and the successes and challenges of engaging adopters to facilitate adoptions.

### 3.1. Theme 1: “Doing Great Adoptions” Starts with an Inclusive, Well-Resourced Application Process and a Skilled Team

The first theme captures the core benefits and challenges of the application process from the shelter team’s perspective. In most focus group discussions, participants were supportive of the current online application process. For several participants, this was the only application or pre-adoption system that they had experienced. Many felt a key benefit of the online application process was that it allowed more time to review and discuss the application to consider the best match, drawing on the expertise of the whole team, and as a result, “it is helping with getting the dog to the right person” (P2:FG3).

Others who had participated in previous adoption processes noted that the current process—although it has increased their workload and required additional time to review and assess applications—has given them greater confidence in their decision-making and in their preparation for the adoption interview stage. This indicates that it “puts us at ease and sets them both [dog and adopter] up for success” (P1:FG5).

In contrast, several experienced staff expressed concern about the loss of the more open conversational, face-to-face adoption philosophy. They viewed adoption as a “belief system” that relies on skills to engage with potential adopters and was akin to providing a counselling service. One participant held the very strong view that, “It’s part of our job to read people as well, you know. Face-to-face contact for all of us is better than reading off a piece of paper” (P2:FG6). This suggests tension between centralising processes and overlooking the interpersonal skills vital to facilitating companion animal adoptions. Overall, however, participants agreed that the process afforded them time to consult with each other, verify the accuracy of the information, and identify a more suitable choice of dog if needed. This provides them confidence in their decisions and strategies for the in-shelter adopter meet-and-greet.

During several sessions, participants emphasised the importance of being prepared for the meet-and-greet process, particularly when the nominated dog was considered unsuitable for the adopter. Several participants stated that reviewing applications and checking out any issues was exercising their “duty of care”. Importantly, it also provided staff with a means to learn more about the adopter and to manage risks associated with potentially complex situations behind the scenes. In contrast, in the following scenario of an on-site walk-in application, there was no opportunity for the back-of-house check, which could have allowed this issue to be resolved less publicly.

*…sometimes they’ll be standing right in front of you (while processing their adoption in the RSPCA database) and all of a sudden there’s a POI, person of interest flag, times three and you go oh umm (chatter) and it does get very awkward because they’re going well, “I own the house, my fencing is appropriate all these boxes are ticked”*

(P2:FG1)

Most of these concerns were easily resolved and did not impede the adoption; however, it may require time on-site to contact an animal welfare law officer, for example, to clarify the concern. Evident throughout these discussions was the pressure of limited time and staff capacity to process applications effectively. This raises concerns about resource equity and the consistency of practice implementation across the shelters, as well as the flow-on effects on staff wellbeing, adopter success, and animal outcomes.

Teams also reflected on public perceptions of the online application process and believed that the public viewed the process negatively, saying, “it is too hard, which I guess it is harder than it was” (P5,1 and 2:FG4). Although these views sometimes shifted after discussions with a staff member, who explained to people that the application process has had positive impacts for adopters and dogs. In these cases, it has “reduced [their] return rates by 70%, because we’re putting the appropriate dog in the appropriate home… we’re setting both the owners and the pet up for success. People kinda go Oh yeah” (P3:FG4). This example highlights the challenge of managing the expectations of those adopting a dog from a shelter while also meeting the staff’s duty of care for the dog and adhering to the organisation’s policies and practices.

Furthermore, several teams agreed that the online application processes had reduced “impulse buying”. The time required to complete an application, submit it, and respond to follow-up questions imposed “thinking time” on the applicants. As P3 says, “The whole online process pushes, as I said, them back into their homes and gives them time to have a think” (P3:FG3).

Accessibility and equity issues affecting unique groups within their communities were also raised by several participants during the discussions. Different teams discussed individuals who may lack access to or expertise with web-based processes. People with no fixed address, renters, linguistically diverse groups, people with a disability and travellers also posed challenges for teams, as forms routinely expect a home address and language proficiency. Participants questioned whether the current online approach discriminated against such groups or was too lenient. In response to the question, “Does the process suit your community?”, one team provided this response.

*Yes and no. (All participants) We were actually talking about it the other day. There are a lot of people with disabilities, who have carers. It is very hard to have conversations [with some of these people] to find out how eligible they are without discriminating. That’s probably been one of the toughest ones. I don’t think you’d find that out from our process of application.*

(P1:FG6)

A couple of the teams raised issues they encountered with people living in rented accommodation in their community who had not sought permission from the owner or rental agent to keep a dog on the premises. Experience with perceived preventable returns made the staff very cautious and more inclined to seek evidence of permission, despite this mandate being removed from the application form (https://rspcaqld.jotform.com/253137993854975?_gl=1*1o5i3ks*_gcl_au*NDI5NzY5MzI2LjE3NjkzOTM5NjA.*_ga*MjQyODEwODg4LjE3NjkzOTM5NjE.*_ga_CH54BHYHTL*czE3NjkzOTM5NjAkbzEkZzAkdDE3NjkzOTM5NjEkajU5JGwwJGgw, accessed 20 February 2026) “. Participants recounted instances where permission was not sought and believed the dogs returned in these scenarios were adversely affected, stating that the dog has “done nothing wrong, but because they’ve (the adopters) fudged their application, the landlord says you didn’t contact me so no you can’t have that here” (P2:FG2).

Evident also was the dilemma individuals and teams faced in balancing firmly held personal views on protecting the dog with the risk of trusting adopters working on the information available to them. One team shared that “other shelters will actually turn people away if they’re not the perfect family. And certain people [staff] have mistrust” (P5:FG6). In contrast, other participants acknowledged that reconciling these competing forces of protection and trust can be difficult; however, giving the dog every chance was always a powerful driver in decision-making.

*We got an application through from a livestock truckie, and the dog was going to live in the cabin. He (the dog) was like really reactive to other dogs and it was probably going to go tits up [sic], but we’ve got virtually no choice we’re staring down euthanasia. Let’s get him (the truck driver) in, let’s see if we like him. Let’s dedicate an hour to really go through everything. He ended up taking him.*

(P4:FG1)

Staff are the interface with the broader community, and the above examples highlight the powerful impact of personal views and experiences on their daily work, as well as the need for professional development and training.

As noted above, most agreed they would accept in-person applications when practicable. One of the larger shelter teams indicated that now they are facilitating “walk-in adoptions a little bit more because we were finding the public has an expectation that they can come in and just adopt an animal” (P1:FG3). The data indicated broad support for a pre-adoption application process, including the flexibility to accept walk-in adoptions, which still required completion of the application form as a useful step in exercising the duty of care for the dogs.

In charting the evolution of the pre-adoption process following COVID-19, it is evident that a more flexible, tailored and conversational approach is re-emerging—one that, as these teams express, is less transactional and rigid, arguing the case that many adoption opportunities might otherwise be missed.

*For RSPCA we need to be communicative and wrap our arms around a situation and be more open to people having an experience, paper and computer systems can’t do that. Are we missing having great opportunities of doing great adoptions across a lot of other RSPCAs?*

(P3:FG6)

Staff discussed limited time and resources as challenges that impacted their capacity to implement the application process effectively. “I feel like it’s a good system, but I feel like we struggle here because we don’t have the staff to properly field the applications” (P1:FG4). This was evidenced in their observations about the organisation process and communication systems. For example, when fielding calls from applicants, who were anxious to learn the outcome of their applications, it was commented that “the only thing we see from the online end is that people will send it (the application form) two or three times. It gives them nothing, apparently. Yes, that’s true, no read receipt or anything” (P1, 2, and 3:FG2). Reportedly, the submissions are sent to a central point and are then forwarded to the relevant shelter. Several participants also acknowledged the impact this could have on prospective adopters.

In summary, this theme highlights several notable sub-themes: the need for process flexibility to provide equitable access for all groups in the local community, the need for sufficient resources allowing time and the breadth of expertise for staff to carry out their jobs effectively, the value of efficient systems to support a responsive application process, and the inherent tension in their roles as carer and adoption advocates. This tension is compounded further by the disjuncture occurring between the ideal state and the reality of “a workable match”. An insightful reflection from one group discussion concerning the application form captured these difficulties in the assessment process and the implicit expectations, personal values, and expectations of those reading the application.

*We say it all the time. If all of us were to fill out an application, none of us would probably look great. We all work full time we have lots of responsibilities (lots of voices in agreement). Like we are quite careful when we are going through applications, to say look, they might be away from the house 10 h a day, but aren’t you away from the house 10 h a day? And we have to give people the benefit of the doubt really.*

(P3 and 4:FG1)

### 3.2. Theme 2: Finding the Right Fit Involves Navigating the Duality of Carer and Advocate Through Honest, Informative Interactions with the Whole Family

It is important to recognise that the meet-and-greet process occurs in a busy public shelter. It involves a unique mix of dogs (who are often not at their best in the shelter environment); prospective adopters who may be excited, expectant, and anxious about taking home a new companion animal; and the shelter staff. These staff members are responsible for the dogs in their care and are striving to find them new homes. This theme explores how staff navigate the duality of carer and advocate through honest, informative interactions with the whole family involved in the prospective adoption. As P2 succinctly stated, “The adoption is what drives us to want to be employed by RSPCA” (the team all murmured in agreement, FG6).

Staff universally agreed that the meet-and-greet was a crucial step in the adoption process. It draws on learnings from the application process; however, as one participant enthusiastically expressed, “the whole meet-and-greet process is huge compared to just the paperwork” (P3:FG3). During these meetings, staff take the information provided in the application form and the conclusions they may have drawn from it and balance these against their observations and information gleaned from the interview and meet-and-greet session. P3 shared that,

*Lots of times you’ll see this nothing application and you’ll think God, can I even bother getting in touch with these people, like they’ve told us nothing. But then they’ll turn up, and the whole thing is magic. They absolutely love the dog and the dog loves them. And they’ll start talking to you, and they’ll tell you so much more than they’ve actually told you in that piece of paper.*

(FG2)

Face-to-face interactions provided the best opportunity to assess whether the dog and adopter were potentially a good match and, when relevant, to evaluate compatibility with any other dogs in the household. Again, emphasising the importance of interpersonal skills. Central to this dynamic and often emotionally charged process was involving the staff members who know “the animals in (their) care and knows them personally” as partners in the adoption (P3:FG1). Teams held that this was fundamental to adoption success. Imperative also was the need to “tailor things to the person (adopter) and the dog, and the person interviewing them, it gives them the best opportunity for a quality outcome. All three parties” (P3:FG1). Likewise, building rapport with adopters and learning more about them and their situations can sometimes lead to unexpected and rewarding outcomes, as illustrated above when a participant recounted her experience, “But then they’ll turn up, and the whole thing is magic” (P3:FG3).

However, some combinations of people and dogs do not work well together. Participants discussed the approaches and tactics they used to navigate these sometimes-tense situations, helping participants recognise that their initial choice might not be the right match for them. Experience had shown staff that steering people in a different direction was often more effective than giving a flat refusal, sharing that, “If you just told someone noooo he’s not good for you, they’re not really going to want to listen” (P3:FG3). Practical experience, however, had shown that if you can engage in conversation and paint a picture of how the dog might “act at first” and describe the ongoing care requirements for that particular dog, often, “when people hear about all the work they are going to have to put in with certain animals, that will just naturally deter them” (P3:FG3). A significant insight into the need for well-honed skills in negotiation and persuasion.

In some cases, people insist on meeting the dog despite the staff’s expressed concerns about suitability. One participant in FG6 said it is often best to allow them to meet the dog if it is safe to do so.

*Kindly show them, they then believe it. Because the little kid wouldn’t leave the dog’s head alone. [..] And then at the end, they accept it, but I would have taken it to the next level, where I say, “I’m very sorry we work for the dog and it’s not the perfect fit, but I will help you find a dog.”*

(P2)

However, there are times when a refusal is warranted. For example, where there is already a dog in the home and during the meet-and-greet, things “go horribly, terribly wrong,” staff argued this was one situation that justified saying “absolutely not. This is not a good match, and it is not going to be good for the dogs” (P3:FG3).

These examples reinforce the importance of meeting and interacting with the dog, involving the entire family and any other dogs in the household. Staff reiterated that, through interactions with potential adopters, they increased their confidence in their ability to identify the “right match”—or to recognise when it is not—and in their willingness to support adopters in finding a dog for their family. The guiding question throughout the adoption process is always, “Can the potential adopter meet the dog’s needs?”

During the discussions, several teams also expressed concern for people’s “weird expectations” of animals and their lack of knowledge about how to interact positively with them (FG3). P4 aptly suggested using this time during the meet-and-greet to “Introduce a new way of thinking as opposed to just telling them how it is,” emphasising the importance of using an “empathetic kind of way and to phrase things in a way that they’re the ones coming to that conclusion. You meet them for a small window of opportunity” (P4:FG3). Another participant offered this as an example of ill-founded adopter expectations, explaining that the potential adopters’ knowledge of the dog was, until the meet-and-greet, based entirely on an online image and its accompanying information. Consequently, when they “come in specifically for Tilly and she doesn’t get along with Shadow, their dog. And then you’re like, well that’s it; this match is out” (P4:FG1).

This scenario does, however, present an opportunity to explain the value of the meet-and-greet and potentially redirect them to another match, reiterating that the meet-and-greet is in everyone’s best interests. At this point, staff “say this isn’t the right fit, but hey, we might have a dog that might be suited for this [your] dog” (P4:FG1). Hearteningly, for staff, their efforts are sometimes acknowledged, which demonstrates the skill they bring to negotiating sometimes challenging relational discussions.

*You know what we get from a lot of people? “It’s nice to know you are thinking of the animal and you’re not just trying to get them out the gate”.*

(P5:FG6)

Several teams discussed the interview and meet-and-greet process at length. They shared numerous cases that demonstrated the importance of being honest and upfront with adopters. “Being honest, I think you need to be honest, like 100%” (P4:FG1). Put plainly, “It’s not sugar coating it” (P1:FG3). It is about the dog and its needs, “talking about their behaviour and health status and what’s required of them” (P2:FG6). Consistently, participants agreed that explaining “what they are looking for as evidence of a potentially good match, or what would be evidence to the contrary” (P1 and 2:FG3) was important. “But not just being honest but explaining” (P5:FG1). These honest and open conversations all teams believed supported reducing their return rates.

When asked about dealing with challenging individuals, staff acknowledged that it can be difficult and that using the application form can be a useful fallback strategy to allow time to consider the proposed adoption.

*You can get really, really pushy people. And it is difficult to deal with them. They think they know it all. And once again, this is where, as I said, the online application is so good. You can bring that into play. You can say you have come in and met the dog, so why don’t you pop home and…fill in the application and send it through…and we will get back to you. That does give you a couple of days’ breathing space. And some people don’t come back in; they change their minds.*

(P1, 2, and 3:FG3)

The application process and the meet-and-greet can overlap. The value for staff in coming prepared to the meet-and-greet is evident; completing the form online and submitting it for review can create space and time to consider the proposed match, whereas with the walk-in, i.e., where people do not submit an application online, they come into the shelter and then choose a dog with the expectation of adopting at that time. This approach does not allow staff much time to review and discuss the proposed match. However, in some circumstances, staff can quickly and confidently ascertain that the match is promising. In response to a question about walk-ins, a team replied, “We do on-the-spot adoptions, we don’t just turn them away” (FG3). Remembering Rocket’s story when a young woman came in and said,

*“me and Mum live on 1000 acres, we have cattle”. And we just went yes, come in here fill out this paperwork and we chatted away to her while she filled in the paperwork, and you could just gauge from her that everything was going well.*

(P1:FG3)

These recounted scenarios and experiences from the day of the focus group discussions offered only brief glimpses into the psychological and physical energy required to work in the shelter. The routine and daily care of the animals, the pressure of negotiating with challenging and impatient people, clashes of values over the provision of good animal care and welfare, and celebrations of success are all part of the work. During one of the sessions, the recording was paused while an adoption was successfully finalised, and the team celebrated together with a high five. However, there are also moments of sadness and frustration when outcomes do not meet staff’s expectations or their hopes for a particular dog. This poignant example captures that experience.

*One dog was a stranger danger alert but once you got to know her, she was fine. This guy adopted her and was told not to get it her face. He said, ‘I know dogs’ and what did he do, straight in her face, and she bit him, not seriously, but she paid the ultimate price.*

(P2:FG4)

At the conclusion of adoptions, staff aimed to provide adopters with guidance on transitioning the dog to their new home and to offer ongoing support if needed. This proved to be quite challenging, as several remarked that during the interview and the meet-and-greet, they fielded many questions; however, when it came time to work on the adoption contract, people were not receptive to further information about settling the dog in or training the dog, as they were too excited about taking home their new dog.

Most shelters provided information in a take-home pack and by email. One team had discussed revising this information to include more fact sheets on a range of common issues, drawing on questions raised by their adopters. The 3-day, 3-week, 3-month infographic is a useful example of getting a message across succinctly (https://www.longmonthumane.org/3-3-3/ accessed on 17 December 2025). Some responses expressed uncertainty about the value or utility of the information provided, indicating that “We do have handouts that we give at adoption, but who knows what happens with those handouts” (FG5 team). Many participants agreed that on the day adopters take their dog home, it is not the best time to expect them to take in information, as they are too excited and focused on the dog. Staff have limited time and opportunity during the meet-and-greet to influence people’s knowledge of dog care and wellbeing. While they provide information, post-adoption may be a more effective time to be proactive and provide support to adopters in those critical first 48 h and when they request support if a need arises. Fundamental to providing effective support is employing adult learning principles and tailoring advice to meet the needs of the adopter and their dog.

There is a wealth of information available, printed and online; however, the perennial challenge is how and when to make it available to such a disparate community of adopters. Teams reiterated that an offer is made for people to contact them if they have questions or want advice once they get the dog home.

Adopters are encouraged to stay in touch. Several participants felt it was important to reassure adopters that they would support them and their dogs for as long as needed. They also reiterated that when a dog is returned, “it is not a failed adoption for that dog, it is a foster experience for that dog. Moreover, you gather really valuable data, as we all know, from the foster homes” (P1:FG7).

*Even if someone does not walk away with an animal, they might still walk away with some education and further knowledge, and I think that is another really important part of the job here that maybe not everyone realises.*

(P4:FG3)

### 3.3. Theme 3: Successful Outcomes Involve Supporting and Educating the Public to Care for and Keep Their Dog

This final theme captures participants’ views and responses regarding the provision of post-adoption support. Building an ongoing relationship with adopters and the community more broadly was seen as a positive step. It is a proactive step in supporting adopters and dogs, while also building trust and confidence in the RSPCA as a source of companion animals and as a place that cares for and actively supports its community in improving animal welfare. P4 captures this obligation well: “We don’t only have a duty to the animals but a duty to the community to educate and spread the message about what we want for dogs” (FG3).

When asked, “What supports a successful adoption outcome?”, participants’ most frequent response was post-adoption support, agreeing that “post-adoption support is a really good thing” (P3:FG3). The type of support raised in the discussion focused on staff’s personal interactions with adopters, for example, offering advice over the phone.

*It helps when they are feeling overwhelmed to have someone to go to, rather than Google where you get all these different answers, and it’s kind of overwhelming. Where if they come to one place (the shelter) they know they’re going to get an answer. They’re going to get help.*

(P3:FG3)

Many participants were aware that the first 24–48 h period is a critical time for new adopters. Support in this period could prevent a dog from being returned by helping new adopters to resolve transition-to-home issues. In one group, they had “talked about that at our meetings, about making a follow-up call a few days after adoption” (FG2). Despite its importance and teams’ willingness to provide this type of support, most shelters offered little structured post-adoption support, constrained by time, expertise, and resource levels.

*We are always happy to help them, but we don’t go out of our way to contact them. One, we don’t have the time to do it, go through all the adoptions and check in on everyone.*

(P1:FG5)

More complex behavioural issues were managed by the behaviour team at Head Office, who communicated directly with the relevant adopter. An issue identified with this centralised support model was the need to improve feedback between state-based teams and the adopting shelter. Staff noted that “We don’t always necessarily know the outcome, but the behaviour team will pop the email responses into the animal file”. Personal contact from the behaviour teams is made in cases where things are not “really working out with the person”, and teams are advised to bring this dog back into the shelter. (P3:FG1).

Several group discussions indicated that participants felt there was a lack of closure, particularly for the dogs they believed might have needed ongoing support to remain in their homes. This sense of a lack of closure might reflect the deep responsibility staff felt for the animals in their care, a responsibility heightened among those who worked closely with them and had formed strong relationships. Length of stay, live-release rate, and return data were used as organisational success metrics; however, staff were not aware of any longitudinal data on adoption outcomes, such as how dogs were doing one, two, or several years post-adoption. During discussions, there was also an implied desire to learn more about long-term outcomes, both for closure and as a learning opportunity to apply and improve their expertise.

Participants in several focus groups expressed a desire to contact people proactively post-adoption, stating that, as they often knew the animal, they could provide support if sufficient resources were available. Put plainly,

*I love helping people over the phone, especially if it is a cat or a dog you’ve been working with. We know them, yes (in chorus) So we’re the ones who can give the most accurate advice on that particular animal. But at the same time as P3 said we don’t have the time to sit there on the phone for twenty minutes or half an hour for every single person who has adopted an animal. I wish we could. I wish we could invest in it, yeah.*

(P3 and 4:FG1)

Involvement in interpersonal contact with new adopters to provide immediate or longer-term support to help people keep their dogs could positively impact job satisfaction among shelter staff and contribute to increased professional expertise. Associated with wanting to provide post-adoption support, a participant in focus group one expressed a personal goal to gain “more behaviour knowledge and to do our own research to do something that is RSPCA specific is something I would definitely like” (P1:FG1).

Implicit and explicit across the dataset was the importance of building relationships with individual adopters and their broader local community. In particular, the regional teams saw this as a way to stay connected with some of the adopted animals and to grow support for their shelter. They understood the value in making adopters “feel like we’re going to be here for them if they need us even in 6 months” (P4:FG1).

Another tangible benefit of long-term relationships was that people became long-term adopters of RSPCA dogs when their initial experience with the shelter was positive. In some shelters, adopters returned to use the shelter’s boarding facilities.

*I think it is important to build that long-term relationship because that customer, if they do lose that dog or that cat, then they are more likely to come back and adopt from us again because they have had that positive experience the entire time. Or we have had them come back and get a second dog…and then they come back to us for boarding (Lots of chatter in agreement).*

(P1, 4, and 5:FG1)

Relationships at the shelter level among staff and volunteers fostered a positive team environment. As demonstrated earlier, the opportunity to draw on team members’ experience and perspectives gave greater confidence in achieving a good match for the dogs in their care. This support was particularly beneficial when dealing with challenging people or making difficult adoption decisions. Elements of this sense of team also included support from the shelter manager and regional managers, with the latter providing flexibility at the local level to tailor processes in the best interests of the animals.

*Adoptions are reliant upon the Manager in adoptions or the Manager sitting above that team to allow teams to take a breath and to really trust that they’ve got this, and that for the animal, to give it a go, to give it a try.*

(P5:FG6)

A concerning aspect of organisational support and relationships related to a perceived or actual lack of consultation or involvement in operational changes that impacted participants’ work and lives. Participants did not always understand the circumstances surrounding the proposed changes. In some cases, offers to contribute may have been overlooked amid competing priorities confronting shelter staff. Comments such as “We weren’t consulted” (for example, when the application process was being reviewed) or reports of a lack of involvement in challenging strategic decisions directly affecting the future of staff and shelters were interpreted by participants as evidence of “not being valued” within the larger organisation.

During discussions on the importance of community relationships and support for shelters, the value of foster carers and volunteers as key resources was raised. In several areas, finding and enlisting foster carers proved difficult. It was suggested that people’s costs, work, or owning other pets are barriers to fostering. There was also misinformation about the costs incurred. From the perspective of would-be foster carers and volunteers, they believed that “You took too long” to reply. Past processes included holding monthly induction morning teas to build relationships and connections with the shelter. Again, discussions indicated that the volunteer and foster programmes were managed at the organisational level. One participant believed the programme “definitely needs to be revamped, our whole programme throughout the State. It’s not good enough. It is not effective enough” (P1:FG1).

Amid the immediacy of daily work in the shelter, these participants, at the conclusion of their session, commented on the importance of their role to provide support to adopters and more broadly to their community to improve animal welfare:

*We have a duty not only to the animals but also to the community to educate and spread the message we want about dogs. Sometimes, what it takes is somebody bringing in their dog to realise it doesn’t want a friend (general agreement), so you just have to show them. It just wants you, it’s fine with that. We have a broader mission than just finding animals homes We have a much bigger mission in terms of educating the community. Changing how the world reacts.*

(P1, P2, and P3:FG3)

## 4. Discussion

Although extensive multidisciplinary research exists on animal rescue organisations, volunteers and shelter workers, research specifically examining the perspectives of shelter workers directly involved in dog adoption processes remains limited and largely unexplored [63]. This study aimed to foreground the voices of RSPCA Qld shelter workers to gain a more in-depth understanding of their daily work experiences on the frontline of dog adoption.

Analyses highlighted that current processes are still evolving since the end of the more rigid, predominantly online COVID-19 process. We found most teams valued the application process, notwithstanding that it increased their workload. Importantly, they indicated that using a structured—though not necessarily rigid—process gave them confidence in this first stage of the adoption matching process. It provided an opportunity to undertake due diligence, and it helped staff balance the tension between protecting each dog’s welfare and finding it a good home. Shelters have a responsibility to ensure the safety of staff, animals, adopters and the broader community. Therefore, a well-designed and managed application process could provide an evidence-based safety net, supporting teams in exercising their professional judgement and managing the risk associated with adopting a dog into a family that is part of the broader community.

It was evident from the focus group data that the adoption interview was the critical stage of the adoption process. Several focus group discussions echoed broader debates among shelter organisations and researchers advocating for an open adoption process that reduces screening and relies less on criterion-based requirements. In contrast, others caution that it may not deliver such benefits [62,64,65,66,67]. However, most teams reiterated the benefits of a structured process over a rigid, criteria-based one, emphasising the need to maintain flexibility to tailor approaches to their shelter and local community. This stance aligns with arguments that balanced processes enabling open conversation and honest disclosure are most effective for negotiating a good match [62,67,68]. Teams unanimously agreed that honest disclosure about the dogs’ needs was paramount, as was the quality of conversation with adopters during the meet-and-greet. Staff viewed the meet-and-greet as the most important step in the adoption process, and involving the person who knew the dog was imperative. An experienced staff member described this part of the matching process as a form of counselling service.

In a recent study on shelter matching programmes, Reese [62] found that many shelter workers are not trained in adoption counselling. Similarly, Irvine [33] argued that shelter workers need additional qualifications and training to undertake their complex, interactive roles. Several participants in this study indicated that they would like access to more training across their different responsibilities. In some teams, staff reported learning mostly on the job from peers and managers.

Notably, two of the teams raised concerns about equity of access for marginalised groups. They believed the application process was inherently discriminatory, as the online format, including specific requirements such as a residential address, was only provided in English, and it relied on a level of digital capability. Evident also was the implicit discrimination in some of the values and opinions expressed by participants regarding people they did not view as good adopters. This discrimination may also extend to access to other adopter and community-based support programmes. 

Ensuring equity of access is multifaceted and complex. Supporting *all* prospective adopters in their efforts to adopt and retain a companion animal requires re-examining accepted policies, practices, and processes through a contemporary lens that values diversity and inclusion [69]. Embedding the One Welfare framework as a governance instrument is advocated by researchers and practitioners as an ethical framework to support shelters in working intentionally to improve “animal welfare, the wellbeing of staff, volunteers and the wider community”, cognisant of the interconnectedness of humans, all other animals and the environment [70,71,72,73]. There is a growing expectation for all animal rescue organisations and animal welfare professionals to embrace diversity, equity and inclusion into their practices and policies [71,74,75,76].

Distinct variations in the approach and implementation of processes were evident across shelters. These variations appeared to be influenced by several factors: individual staff members’ personal views on what constitutes a good match, how they engage with potential adopters, shelter teams’ culture and leadership style, as well as organisational resource allocation and the local community context [63]. Protopopova et al. [77] discuss the complex interaction of similar factors in their study about a meet-and-greet intervention across several shelters. These findings also align with many reported research findings on companion animal adoption and relinquishment, including staff wellbeing, volunteers, and shelter practices and programmes [64,78,79,80].

Equally valued in supporting good adoption outcomes is post-adoption support. However, the individual shelters in this study reportedly offered limited post-adoption support. Although staff wished to call and follow up with new adopters, limited time and resources were cited as barriers. Shelter teams invited all adopters to contact them with any questions or concerns, and the centralised behaviour team followed up with dogs identified as having behavioural issues. Protopopova and Bollen [78] questioned the efficacy of this strategy. Given the limited engagement from many adopters, post-adoption, it raises a question of investigating a more proactive and differentiated approach. Email and social media were described as convenient modes to receive feedback on the dog’s post-adoption status. In many shelters, community events, such as the annual Million Paws Walk, were also highlighted as a way to maintain contact with former adopters, strengthen relationships, and build support in their communities. Reese [80] in *Strategies for Successful Animal Shelters*, suggests that building community relations, being present in the community, providing education, fostering volunteer programmes, and bringing people into the shelter are strategies to improve wellbeing outcomes for animals and people.

The benefits of post-adoption support in helping people and dogs remain in their homes are widely recognised in the research [64,78,79]. Furthermore, it is argued that support provided within the first 24 to 48 h post-adoption can be highly effective [81,82]. It can be inferred from the focus group discussions that the availability of post-adoption support at RSPCA Qld shelters warrants review. Similar findings were reported in a 2006 survey by Burch and colleagues [82], which indicated that the area requiring development and improvement in shelter adoption processes was adoption follow-up, along with the training needed to conduct it effectively. However, shelters could consider a more proactive approach and instigate sending an email to all adopters within the first 48 h, offering support, providing targeted follow-up phone calls with adopters, and using experienced foster carers or other upskilled volunteers to provide support or advice [78,83].

In reflecting on the analyses and the three themes generated, it is important to note that the significance of relationships and communication was evident across all themes. These were fundamental to the healthy functioning of teams and their individual members, providing professional and social support that can protect staff working in an environment characterised by high turnover, loss of expertise and experience, and a high risk of burnout and traumatic stress [34,35]. Building rapport and establishing strong relationships with prospective adopters was also key to ensuring a good match. At the community level, relationships and communication play an important role in maintaining support for and trust in RSPCA Qld as an organisation that provides care and protection for animals in the local community. Participants and adopters identified gaps in communication and feedback processes with the Head Office, particularly in seeking staff input on policy and process discussions that directly affect their work in shelters and timely responses to prospective adopters and shelters when application forms are submitted. Drawing on people’s experiences and insights is essential to improving policies, processes and outcomes. It also demonstrates that their insights and contributions, as individuals with frontline experience, are valued. These local observations raise questions about the level and effectiveness of intra-organisational consultation on matters of policy and process and the responsiveness to potential adopters. Communication in the broadest sense ranged from general engagement with visitors—marketing the shelter and providing information about the shelter’s work or a particular animal—to more in-depth, nuanced discussions with adopters.

A strength of this study is that it highlights the complex, varied daily work of caring for animals, preparing them for adoption, and negotiating the best fit between the animal and the adopter. Although not generalisable, this small sample underscores the value of being on the ground with shelter workers, as qualitative research can capture their stories and provide an in-depth understanding of what is working well, what challenges exist, and what they need to improve welfare adoption outcomes for the animals in their care. This on-the-ground approach can road-test the authenticity of quantitative data. It can also help identify opportunities and gaps to better support shelters.

## 5. Limitations

This qualitative study captures the insights and experiences of RSPCA Queensland shelter staff about adoption processes in their respective shelters. While it provides rich, detailed data on adoption processes across several shelters, caution is warranted when generalising the results, given that it was a small sample within a single rescue organisation. The study highlighted a large variation in process implementation, staff experience and training, and shelter resourcing levels. It is noted that the adoption process is a complex, dynamic interaction among dogs, staff, and potential adopters, influenced by many factors that can affect adoption outcomes.

## 6. Conclusions

Shelter teams are central to successful dog rehoming. Results indicate that shelter workers value processes that provide structure and support for implementing adoption procedures, while also allowing flexibility to tailor these processes to local needs to achieve the best outcomes for both dogs and prospective adopters. Involving those who know the dogs best is critical to negotiating a good match between dogs and prospective adopters. Open and honest conversations with adopters about the dogs’ needs and how they might be met, as well as about adopters’ expectations, are essential. Ongoing support for adopters post-adoption was considered important for dogs remaining in the home. In short, adoption is a team effort drawing on a range of staff perspectives and expertise. Staff identified time and resource constraints as challenges to implementing all the adoption processes effectively. This was particularly the case with the limited local provision of post-adoption support. Successful adoptions, it was agreed, could be more effective if teams were better resourced, offered tailored training, and supported by efficient, less centralised, more inclusive adoption procedures.

## Data Availability

The data collected for this study are not publicly available due to the ethical approval of participant informed consent, which assured them that all personally identifiable information would be removed before analysis and prior to publication.

## Appendix A

**Study 3 Group Discussion Guide and Questions**

**Outline of the session**

Thank you for giving up your time to come today. My name is (moderator). The discussion groups are part of my research into dog adoption and relinquishment.

Your experiences are invaluable to this research, as few published studies investigate the experiences of shelter staff (or volunteers) involved in dog adoption and post-adoption support.

I have some questions to guide the discussion. We will work through three topics: the application process, the adoption interview and taking the dog home, and follow-up and post-adoption support. Please share anything else you believe is crucial to this topic as we progress.

I will take notes during our discussion; however, I would like to record the session to ensure we capture all the valuable insights. If a scribe is engaged for the larger sessions, this person will be introduced, and participants will be informed that he/she is not one of the researchers and will not have access to any data collected. He/she is bound by the same confidentiality as session participants. The recordings will be used solely for research purposes and securely stored on the University of Queensland data management site. Today’s discussions are strictly confidential; only the research team can access the recordings. Is everyone comfortable with this being recorded, and can I confirm you have signed the consent forms?

During our discussions, please let everyone share their views. It would be helpful if only one person at a time spoke so the recording would be clearer. Please join in when you have something to say. I will not go around the group for every question, but I would greatly value hearing your views. It’s OK to disagree or proffer an alternative view. Please respect the opinions of others. Everything we share today should be confidential and not shared with people outside the group. Your privacy and the confidentiality of our discussions are important.

Remember that your participation is voluntary, and you can withdraw at any time.

So, I hope you will feel comfortable sharing your experiences and observations about dog adoption. The session will last about an hour. Please help yourself to tea and coffee.

Are there any questions before we start?

**Discussion Questions/topics**

*Introduction*

COVID-19 had a significant impact on everyone. It was the impetus for RSPCA Qld to adapt its business model and continue to care for all the animals in its care. One of the interviewees in study 2 adopted Susie, the dog he acquired during ‘Clear the Shelters’. Their journey is an insightful story about patience and building mutual trust. She is still with him.

*Discussion*

**Topic 1: Adoption application process**

• With that in mind, what are your reflections on how COVID-19 has changed your dog adoption application processes?
○ Have you tailored the application processes to suit your community?
○ Do you believe it is working well?
○ What other changes would you make?
○ What are the biggest challenges for you in your role?

**Topic 2: The adoption interview and taking the dog home**

• In reflecting on your interactions with potential adopters, do you think your processes facilitate a good outcome for the dogs and adopters?
○ What do you think contributes to a successful outcome?
○ Are there things that would better support you or your team during this process?

**Topic 3: Post adoption follow-up and support**

• Could you tell me about contact with owners once they take the dog home?
○ The types of support are you able to provide to adopters
○ Tell me about your successes
○ Tell me about your challenges.

**Closing questions and discussion**

• Are there other comments you would like to make about your insights into improving the long-term outcomes for our adoptions?

*Thank participants*
